# Prenatal diagnosis of exomphalos and prediction of outcome

**DOI:** 10.1038/s41598-021-88245-0

**Published:** 2021-04-22

**Authors:** K. Nitzsche, G. Fitze, M. Rüdiger, P. Wimberger, C. Birdir

**Affiliations:** 1 Department of Obstetrics and Gynecology, University Clinic of Carl Gustav Carus Dresden, Technische Universität Dresden, 01307 Dresden, Germany; 2 Department of Pediatric Surgery, University Clinic of Carl Gustav Carus Dresden, Technische Universität Dresden, 01307 Dresden, Germany; 3 Department of Pediatrics, University Clinic of Carl Gustav Carus Dresden, Technische Universität Dresden, 01307 Dresden, Germany; 4 Saxony Center for Feto/Neonatal Health, Technische Universität Dresden, 01307 Dresden, Germany

**Keywords:** Anatomy, Diseases, Gastrointestinal system

## Abstract

The aim of this study was to detect a parameter for predicting prenatal complications or postnatal surgical options after detecting a fetal exomphalos. This was a retrospective analysis of prenatal diagnosis and outcome of fetuses with 41 cases of exomphalos in between 2007 and 2017, considering the measurement of ratios. The 41 fetuses with exomphalos were examined, 34 cases (82.9%) with karyotyping and 16 cases (39%) with an abnormal karyotype. Outcome of 39 cases showed 6 abortions (15.4%), 15 terminations (38.5%), an intrauterine fetal death (2.5%) and 17 alive babies (43.6%), which were grouped in two: small exomphalos (n = 6, 35.3%) and big exomphalos (n = 11, 64.7%). Maximal diameter of exomphalos/abdomen circumference-ratio (EDmax/AC-ratio) with a cut-off of 0.24 showed a better predictive value of postnatal primary closure. Exomphalos is correlated with abnormal karyotype. EDmax/AC-ratio gives the best prediction for postnatal primary closure of the defect.

## Introduction

The most common abdominal wall defect of the newborn is exomphalos. Exomphalos is an abdominal wall defect of the umbilical ring. The intra-abdominal organs herniate in the umbilical cord, which is covered by a membrane of peritoneum and amnion. The bowel fails to rotate back into the abdomen in the embryological period between 6 to 11 weeks of pregnancy in which this herniation is physiological^1^. The incidence of exomphalos is 1.9–2.5 in 10,000 live births^2–4^, but 1 in 1100 pregnancies^5^. It is associated with genetic defects and other fetal anomalies, and the most common association is with Trisomy 18^6,7^. It is seen in many syndromes like Pentalogy of Cantrell, body stalk anomaly, and Beckwith-Wiedemann syndrome^8^. Many of these pregnancies are lost due to combined chromosomal and structural fetal anomalies^9^. Besides these anomalies, the size of the exomphalos is an important factor for the outcome of the fetus and postnatal therapy options. There are not any uniform definitions for the size of exomphalos. Different authors define the size of exomphalos as a defect varying from 4.5 to 5 cm diameter in size with liver enclosed^10^ or as more than 75% of the liver in the defect^11^. A large exomphalos is associated with small abdominal cavity and decreased possibility of primary closure of the defect postnatally^10^.

The aim of this study was to find a prenatal parameter to be able to predict possible prenatal complications, or postnatal surgical options. This parameter will allow the fetal medicine specialist together with pediatric surgeons and neonatologists an improved counseling of the parents and to determine the timing of delivery and therapy.

## Materials and methods

The prenatally diagnosed exomphalos cases were examined once again retrospectively. These patients were scanned in our unit between 2007 and 2017. The scans were performed using two Voluson E8 machines (General Electric) and an Epiq 7 machine (Philips, Amsterdam, Holland) by consultants of fetal medicine (certified by Fetal Medicine Foundation (FMF) in London or German Association for Ultrasound in Medicine (DEGUM)). A complete anomaly scan was performed to diagnose or exclude other associated defects. An invasive genetic testing was offered to these patients after the diagnosis.

After diagnosing exomphalos, a counseling has been offered to the couples. These patients delivered in our hospital. The department of pediatric surgery performed the surgical procedures. The digital documentations of the patients were examined to extract the data.

**Figure 1:**
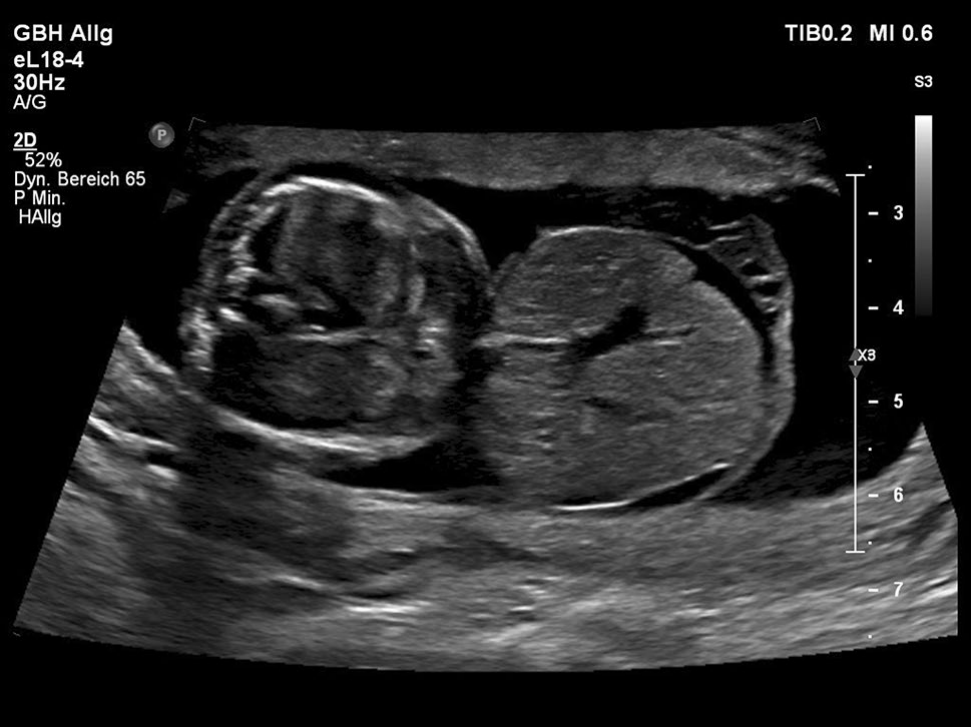
Big exomphalos with liver herniation.

**Figure 2:**
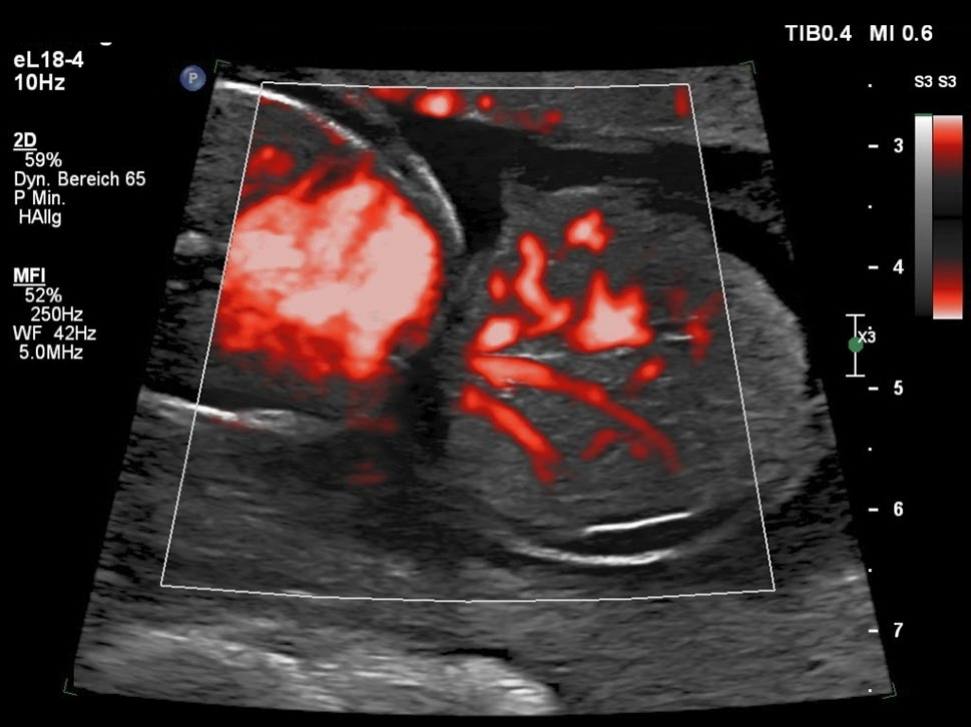
Big exomphalos with liver herniation.

**Figure 3:**
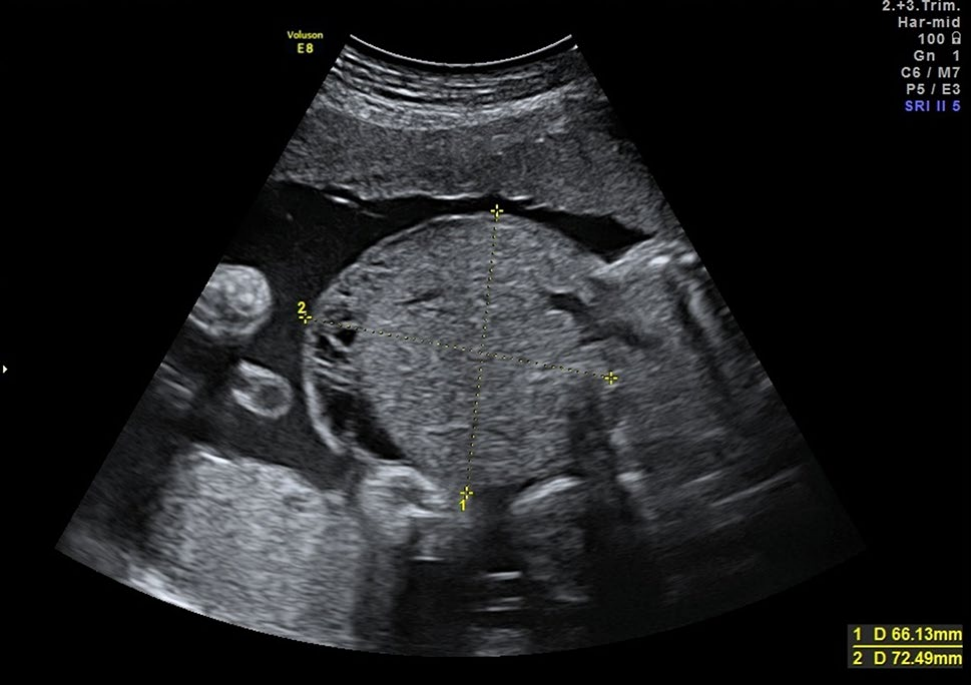
Measurement of apED and EDmax.

The fetuses with exomphalos were classified according to the scan reports, other anomalies and chromosomal aberrations. Regular ultrasound scans were offered every 4 weeks to measure the fetal biometry. The abdominal circumference was measured using two linear measurements: transverse abdominal diameter and anterior–posterior abdominal diameter (APAD). The measurements and pictures were saved digitally in Viewpoint documentation system (General Electric). The cases were divided in two groups: small Exomphalos (diameter of exomphalos (ED) below 4.5 cm) without liver herniation and big Exomphalos (ED above 4.5 cm) with liver herniation^10,12^ (Figs. 1 and 2). Using one of the saved images after 30 weeks of pregnancy retrospectively, the same examiner performed the following measurements: circumference of exomphalos (EC), anterior to posterior diameter of exomphalos (apED) and maximal diameter of exomphalos (EDmax) (Fig. 3). The following cut-off values were obtained using the fetal abdomen circumference (AC) and head circumference (HC): ED/AC as previously published by Kleinrouweler et al*.*^13^; apED/AC as previously published by Fawley et al*.*^14^; EDmax/AC and EDmax/HC as previously published by Montero et al*.*^15^. These cut-off values are then used to predict the possibility of a primary closure of the fascia postnatally. The sensitivity, specificity and positive predictive value (PPV) of these values were assessed. The best gestational age of measurement was between 30 and 32 weeks to make a prediction about postnatal primary closure. Excel was used (Microsoft Corporation, Redmond, Washington, USA, 2010) for the statistical analysis. The ethics committee of the Technical University of Dresden, Germany approved this study (BO-EK-66022020, 11 March 2020).

### Ethical approval

The ethical committee of the Technical University of Dresden has approved the study.

### Informed consent

We confirm that all methods were carried out in accordance with relevant guidelines and regulations. There are no known conflicts of interest associated with this publication and there has been no financial support for this work that could have influenced its outcome. The manuscript has been read and approved by all named authors. An informed consent from all participants were taken.

## Results

Fetal exomphalos was diagnosed in 41 singleton pregnancies with a prevalence of 1.6/1000 between 2007 and 2017 among 25,107 delivered babies. The median maternal age was 31 (between 19 und 47) (Table 1) and the first scan performed was between 11 + 3 and 37 + 0 weeks of pregnancy. In 34 cases (82.8%), an amniocentesis was performed, whereas 7 patients declined genetic testing (17.2%). Among 34 cases with amniocentesis, 18 cases were with a normal karyotype (52.9%) and 16 cases with an abnormal karyotype (47.1%), respectively. Among the cases with an abnormal karyotype, 9 fetuses were diagnosed with Trisomy 18 (26.5%), 1 fetus with Trisomy 13 (2.95%), 1 fetus with Trisomy 21 (2.95%), 3 fetuses with Turner Syndrome (8.8%), 1 fetus with Triploidy (2.95%) and 1 with Klinefelter Syndrome (2.95%).

**Table 1:** Maternal characteristics and outcome of cases with exomphalos.

Parameter		Exomphalos (N = 41)
Age (years)	Mean	31 (19–47)
First examination (weeks of pregnancy)	Mean	18 + 3 (13 + 1–37 + 0)
Karyotyping	Not performed	7 (17.2%)
	Performed	34 (82.8%)
	Normal	18/34 (52.9%) 13 alive
	Abnormal	16/34 (47.1%) 14 alive 20 termination and intrauterine fetal death
Other anomalies		26 (63.4%)
Outcome	Alive	17
	Intrauterine fetal death	1
	Abortion	6
	Termination	15
	Lost	2
Delivery (weeks of pregnancy)	Mean	35 + 6 (31 + 1–40 + 0)
Mode of delivery	Vaginal	3 (17.6%)
	C-section	14 (82.4%)
Weight of the newborn	Mean	2665 (790–3660)
Weight of the newborn (%)	Mean	35. percentile (1–85)

According to the ultrasound parameters, 15 cases (36.6%) had no other anomalies and 26 fetuses (63.4%) had other anomalies seen. In 12 cases without any other anomalies, a karyotyping was performed and 11 of these fetuses (91.7%) had a normal karyotype and 1 fetus had Trisomy 21 (8.3%). In 22 cases with anomalies apart from exomphalos, a karyotyping was performed, and 7 fetuses had a normal karyotype (31.8%) and 15 fetuses had abnormal karyotype (68.2%).

In 18 cases with exomphalos, a first trimester screening was performed. In 10 cases (55.6%) there were other anomalies and a nuchal translucency above ≥ 3.5 mm seen. 9 of these cases had a genetic testing and none of them had a normal karyotype.

Among 26 cases with other anomalies (63.4%), there were cardiac (30.8%), skeletal (29.9%), cerebral (15.4%) and urogenital system (11.5%) anomalies and diaphragmatic hernia (7.7%), Pentalogy of Cantrell (7.7%) and fetal growth restriction (IUGR) (3.8%).

It was possible to retrieve the outcome of 39 pregnancies, and 2 patients delivered elsewhere. There were 17 deliveries (43.6%), 6 abortions (15.4%), 15 terminations of pregnancy (38.5%) and one intrauterine death (2.5%). The prevalence of exomphalos among live births was 6.8/10,000.

In 14 cases of deliveries, a postnatal karyotyping was performed with a result of a normal karyotype. 3 of these women refused a prenatal karyotyping.

One newborn died after 2 days of delivery due to Pentalogy of Cantrell and related anomalies.

The group of cases with live births was divided in 2 groups using the ultrasound measurements: 6 fetuses (35.3%) with small exomphalos (EDmax below 4.5 cm) and 11 fetuses (64.7%) with big exomphalos (EDmax above 4.5 cm).

In 5 cases, a therapy with corticosteroids was given for preventing infant respiratory distress syndrome.

The deliveries were between 31 + 1 and 40 + 0 weeks of pregnancy (median 35 + 6 weeks) with 14 cases of c-section (82.4%) and 3 cases of spontaneous delivery (17.6%) which had small exomphalos. The median birthweight was 2.665 g (between 790 and 3.660 g).

The surgical repair of the defect was possible as a primary closure in 10 cases (58.8%) and in 6 cases (35.3%) fascia displacement procedure was performed. In one case (5.8%), a silo was used due to the size of the defect and the baby died at the second day after delivery. The cases with primary closure had all small exomphalos. Among the babies with big exomphalos, 4 of them (36.4%) had a primary closure, 6 fascia displacements (54.5%) and one needed silo placement (9.1%). The associated anomalies and karyotype aberrations of 17 fetuses with post-natal repair are shown in Table 2.

**Table 2:** The associated anomalies and karyotype aberrations of 17 fetuses with post-natal repair.

Case	Size of exomphalos	Karyotype	Prenatal associated anomalies
1	Big	46, XY	None
2	Big	46, XY	None
3	Big	Not known	Clubfoot
4	Big	46, XX	Pentalogy of Cantrell
5	Big	46, XY	None
6	Big	46, XX	None
7	Big	46, XY	None
8	Big	46, XY	None
9	Big	46, XX	None
10	Big	46, XY	None
11	Big	46, XY	Pentalogy of Cantrell
12	Small	46, XX	None
13	Small	46, XY	None
14	Small	46, XY	Ventriculomegaly
15	Small	Not known	None
16	Small	Not known	None
17	Small	46, XX	None

The sonographic cut-off values of 0.75 for ED/AC^13^, 0.26 for apED/AC^14^, 0.24 for EDmax/AC and 0.21 for EDmax/HC^15^ were used in our collective to predict a primary closure postnatally. These cut-off values were previously published. In the group of babies with small exomphalos, it was possible to predict a primary closure in all cases (Table 3).

**Table 3:** Prediction of big exomphalos without primary closure.

	Ratio	Primary closure	No primary closure		Sensitivity %	Specificity %	PPV %	NPV %
			Fascia displacement	Silo				
ED/AC	≥ 0.75	1	4	1	71.4	75.0	83.3	60.0
	< 0.75	3	2	0				
apED/AC	≥ 0.26	1	2	1	42.9	75.0	75.0	42.9
	< 0.26	3	4	0				
EDmax/AC	≥ 0.24	1	6	1	100	75.0	87.5	100
	< 0.24	3	0	0				
EDmax/HC	≥ 0.21	1	3	1	57.1	75.0	80.0	50.0
	< 0.21	3	3	0				

ED/AC: diameter of exomphalos/abdomen circumference; apED/AC: anterior to posterior diameter of exomphalos/abdomen circumference; EDmax/AC: maximal diameter of exomphalos/abdomen circumference; EDmax/HC: maximal diameter of exomphalos/head circumference; PPV: positive predictive value; NPV: negative predictive value; ratio in mm.

In the group of babies with a big exomphalos (n = 11), it was possible to diagnose the big exomphalos in 6 cases (54.5%) with an ED/AC cut-off ≥ 0.75. Among these babies, 1 had a primary closure; 4 had fascia displacement and 1 silo placement. The ED/AC ratio fell below the cut-off of 0.75 in 5 cases. 3 of these babies had primary closure and 2 fascia displacement.

A cut-off of 0.75 for ED/AC ratio reached a sensitivity of 71.4% and a specificity of 75.0% in our collective to predict a primary closure. The cut-off values of apED/AC and EDmax/HC gives a sensitivity and specificity of 42.9% and 57.1%, respectively. A cut-off value < 0.24 for EDmax/AC to predict a primary close has a sensitivity of 100% in our collective, 75% specificity, 87.5% PPV and 100% NPV.

## Discussion

Exomphalos is one of the most commonly seen abdominal wall defects of the fetus with an incidence of 1.9–2.5/10,000 births^3,4,16^. Our collective showed a prevalence of 6.8/10,000 for exomphalos. This higher prevalence may be because of the higher number of referrals to our fetal medicine department.

Prenatal diagnosis of an exomphalos implicates a poor outcome of the fetus due to common correlation with chromosomal abnormalities up to 28–69%^5,7,13,17,18^, and the most common chromosomal abnormality seen is Trisomy 18^6,7^. A combination of a NT measurement above 3.5 mm and exomphalos at first trimester was seen in 41% of chromosomal abnormalities^3^. An exomphalos with a normal karyotype is associated with other anomalies up to 26–89%^5,7,13,19^. There is also an association with Beckwith-Wiedemann Syndrome and Pentalogy of Cantrell^7,20^. The most common chromosomal abnormality seen in our collective was Trisomy 18. An exomphalos combined with other structural abnormalities and a NT measurement above 3.5 mm at first trimester of pregnancy did not show any chromosomal abnormalities in our collective. Due to the high number of chromosomal abnormalities and structural anomalies, the rate of abortions and terminations were seen up to 66%^7,13,15,21^ and the rate was 53.9% in our collective (15,4% missed abortion and 38.5% termination).

The mode of delivery is dependent on the size of exomphalos. The babies with small exomphalos were delivered normally and the babies with big exomphalos with c-section to prevent the perforation of defect^7,13,15,22^. The closure techniques of the defect are the conservative and operative methods, where again the size of the defect plays an important role^23^. All the babies with a small defect were able to be operated with a primary complete closure in our collective. The measurements of the size of exomphalos can be compared to the biometric measurements of the fetus to be able to predict the therapy options such as primary complete closure of the defect, conservative method or multiple operations. Kleinrouweler et al*.* could predict a primary closure of the defect with a sensitivity and specificity of 100% using the cut-off values of 0.57 till 0.75 for ED/AC ratio where a cut off ≥ 0.75 could exclude the possibility of a primary closure^13^. This method showed a sensitivity of 71.4% and specificity of 75.0% to predict a primary closure in our collective. Two out of five fetuses with an ED/AC ratio below 0.75 could not be operated with a primary closure and one of six fetuses with an ED/AC ratio ≥ 0.75 could have a primary closure.

Fawley et al*.* used a cut-off value of 0.26 for apED/AC ratio to predict a primary closure of the defect with a sensitivity of 100%, specificity of 88.9% and PPV of 85.7%^14^. Using this value, we could reach a sensitivity of 42.9%, specificity of 75.0% and a PPV of 75%, respectively in our collective.

Montero et al*.* examined EDmax/AC and EDmax/HC ratios^15^. They found that an EDmax/HC cut-off ≥ 0.21 could exclude the possibility of a primary closure with a sensitivity and specificity of 84.6% and 58.3% respectively. Using an EDmax/AC ratio, a cut-off ≥ 0.24 gave a sensitivity and specificity of 83.3% and 58.3%, respectively. Our collective showed a sensitivity of 57.1% and a specificity of 75.0% for EDmax/HC ratios, and a sensitivity of 100% and a specificity of 75.0% for EDmax/AC ratio. All of the three fetuses with a big exomphalos and EDmax/AC below 0.24 had a primary closure postnatally with a PPV of 87.5% and NPV of 100%.

The cut-off value of 0.24 for EDmax/AC ratio was the strongest among the others to predict a primary closure with big exomphalos.

The limitation of this study was the retrospective analysis and small number of cases. An accurate and better prenatal diagnosis of exomphalos is possible with measurement of ratios for exomphalos. Using these tools, it is possible to predict primary closure versus non primary closure and plan the postnatal procedures to improve the outcome of these babies.
